# Co-circulation of influenza A(H1N1), A(H3N2), B(Yamagata) and B(Victoria) during the 2017−2018 influenza season in Zhejiang Province, China

**DOI:** 10.1017/S0950268820000412

**Published:** 2020-02-14

**Authors:** Haiyan Mao, Yi Sun, Yin Chen, Xiuyu Lou, Zhao Yu, Xinying Wang, Zheyuan Ding, Wei Cheng, Dan Zhang, Yanjun Zhang, Jianmin Jiang

**Affiliations:** Zhejiang Provincial Centre for Disease Control and Prevention, Hangzhou, Zhejiang, People's Republic of China

**Keywords:** Co-circulation, influenza, influenza-like illness, phylogenetic, vaccination

## Abstract

Influenza is a major human respiratory pathogen. Due to the high levels of influenza-like illness (ILI) in Zhejiang, China, the control and prevention of influenza was challenging during the 2017–2018 season. To identify the clinical spectrum of illness related to influenza and characterise the circulating influenza virus strains during this period, the characteristics of ILI were studied. Viral sequencing and phylogenetic analyses were conducted to investigate the virus types, substitutions at the amino acid level and phylogenetic relationships between sequences. This study has shown that the 2017/18 influenza season was characterised by the co-circulation of influenza A (H1N1) pdm09, A (H3N2) and B viruses (both Yamagata and Victoria lineage). From week 36 of 2017 to week 12 of 2018, ILI cases accounted for 5.58% of the total number of outpatient and emergency patient visits at the surveillance sites. Several amino acid substitutions were detected. Vaccination mismatch may be a potential reason for the high percentage of ILI. Furthermore, it is likely that multiple viral introductions played a role in the endemic co-circulation of influenza in Zhejiang, China. More detailed information regarding the molecular epidemiology of influenza should be included in long-term influenza surveillance.

Influenza is responsible for substantial human suffering, illness and death. Of the influenza A, B and C subtypes, influenza A and B are the major cause of epidemics and pandemics worldwide. Annual epidemics occur throughout most parts of the world. Between September 2017 and January 2018, influenza activity was reported in many regions, with co-circulating influenza A(H1N1)pdm09 (short for H1N1), A(H3N2) (short for H3N2) and influenza B viruses (short for B virus), as reported by the World Health Organization (WHO) Weekly Epidemiological Record [1]. Monitoring influenza-like illness (ILI) is considered one of the present global standards to identify the patterns of influenza epidemics, which are essential for the yearly planning of prevention and response activities. In Zhejiang Province, China, it was estimated that ILI accounted for a significant percentage of the total number of outpatient visits, compared to the contemporary influenza seasons from September 2015 to January 2016 and September 2016 to January 2017 (Fig. 1a; Supplementary Table S1). This is based on the ‘National Influenza/Avian Influenza in the Human Surveillance Information System’ from week 36 in 2017 to week 12 in 2018 (2017/18). This system is one of the main constituents of China FluNet, which is a nationwide influenza surveillance network that has been in place since 2000. The function of this network includes ILI monitoring, severe respiratory disease surveillance, outbreak surveillance and viral surveillance [2]. Comparisons were made for the same influenza seasons from 2015 to 2016 (2015/16) and from 2016 to 2017 (2016/17) based on ILI monitoring in the network. The 2017/18 season was a rare and challenging situation for disease control and prevention (Fig. 1a; Supplementary Table S1).

**Fig. 1.**
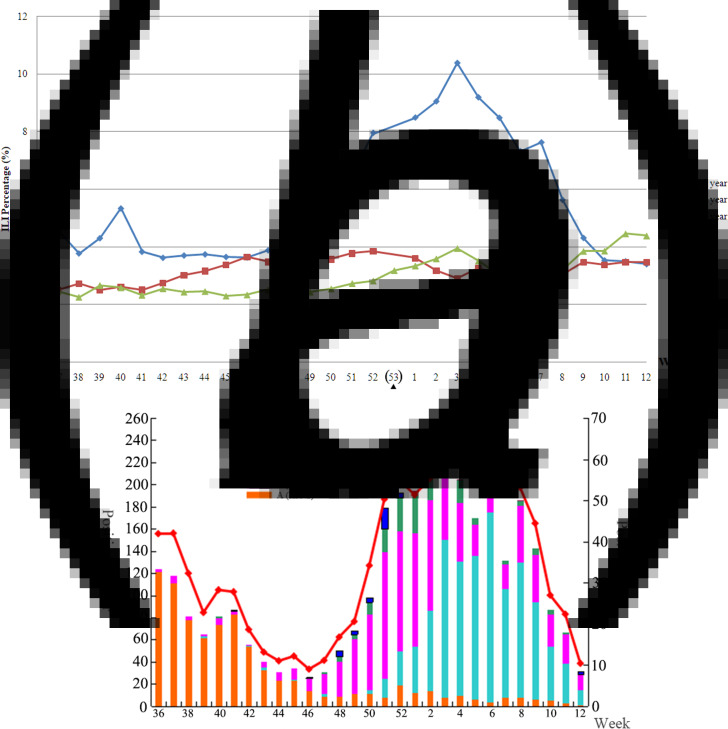
General information during the influenza season. (a) Percentage ILI for the period from week 36 in 2015 to week 12 in 2016, week 36 in 2016 to week 12 in 2017 and week 36 in 2017 to week 12 in 2018. Although there was week 53 in 2015, since there were only 52 weeks in both 2016 and 2017, we listed the ILI percentage for week 53 in 2015 with a black triangle. (b) Positive numbers (bar) and rates (curve) for different influenza strains from week 36 in 2017 to week 12 in 2018.

This study identifies the clinical spectrum of illness related to influenza and describes the strains of influenza that were circulating during the 2017/18 outbreak period in Zhejiang Province, China. These data are invaluable in planning influenza control measures. In particular, the phylogenetics of circulating influenza strains was analysed using sequences that represented different geographic regions in Zhejiang Province. We identified mutation sites in the genomes and identified biological functions related to amino acid changes. The phylogenetic relationships with other classic influenza clusters were primarily determined based on the hemagglutinin (HA) gene.

This study was approved by the ethics committee of the Zhejiang Provincial Center for Disease Control and Prevention (CDC), China. The current WHO definition of ILI is an acute respiratory infection with a measured fever of ⩾ 38 °C and cough with onset within the last 10 days [2]. According to the network, 16 hospitals scattered throughout 11 cities/areas in Zhejiang Province were the surveillance sites for ILI. The location of 16 surveillance sites in Zhejiang Province with a total outpatient visit number on ILI for each hospital in the past 3 years is presented in supplementary materials (Supplementary Fig. S1, Supplementary Table S2). Patient epidemiological data were collected by the local CDC staff and reported to the Zhejiang Provincial CDC and China CDC. Sample collection from 2017/18 was performed according to the WHO's Global Epidemiological Surveillance Standards for Influenza (https://www.who.int/influenza/resources/en/). Viral isolation and identification were carried out in accordance with the WHO's standard protocols [2].

To understand the spatiotemporal distribution of ILI, positive sample rates and the proportion of influenza B in all positive samples in prefecture-level cities, heat maps were prepared using R statistical software, version 3.5.0 (R Foundation for Statistical Computing, 2018). Heat maps were used to: (1) analyse the spatiotemporal series of ILI in outpatient and emergency patients in prefecture-level cities; (2) analyse the spatiotemporal series of laboratory surveillance results of influenza-positive sample rates in prefecture-level cities; (3) determine the proportion of influenza B in all positive samples in prefecture-level cities.

In order to investigate the molecular characteristics of influenza during 2017/18, samples for viral sequencing were chosen randomly from routine surveillance. Viral RNA was extracted using an RNeasy Mini Kit (Qiagen, Germantown, MD, USA) according to the manufacturer's instructions. The viral RNAs were sent to BioGermBio-tech Company (Shanghai, China) for whole genome sequencing. All virus sequences have been deposited in the Global Initiative on Sharing All Influenza Data (GISAID) database (EPI1229388–EPI1229680). All eight segments from each type of influenza were assembled and aligned along with additional sequences downloaded from GenBank using Geneious 10.2.2 (https://www.geneious.com/). Variant positions in the nucleotide and amino acid sequences were checked by Geneious 10.2.2. The phylogenetic analyses were performed using the maximum composite likelihood as a model of nucleotide substitution, and the neighbour-joining method to conduct the phylogenetic tree by MEGA ver. 7.0.14 (http://www.megasoftware.net/) based on the HA segment. The bootstrap value was set as 1000 for statistical analysis.

From week 36 of 2017 to week 12 of 2018, there were 280 920 cases of ILI, which accounted for 5.58% (280 920/5 034 063) of the total number of outpatient and emergency patient visits. This ILI ratio varied between week 36 and week 45, increased from week 46 (3.63%) to peak in week 3 of 2018 (10.37%), and then gradually returned to 3.4% in week 12 of 2018 (Fig. 1a). The average percentage of reported ILI from the 16 surveillance sites during 2017/18 (5.58%) reached higher levels than that reported in 2015/16 (2.99%) and 2016/17 (3.20%) (Supplementary Table S1). The proportion of ILI in outpatient and emergency patients was relatively high from week 1 to week 7 in 2018 and from week 36 to week 41 in 2017, especially in Ningbo and Zhoushan (Supplementary Fig. S2a).

A total of 8300 samples were used for influenza virus detection during the 2017/18 influenza season. Of these, 35.9% (2980/8300) were positive for influenza, including 471 samples of (15.8%) H3N2, 1149 samples of (38.6%) H1N1, 1349 (45.3%) samples of B virus and 10 samples with (0.3%) co-infection. No human H7N9 infection was detected. Samples from patients between the ages of 2 and 67 years old co-infected with influenza A and B were detected between week 36 of 2017 and week 12 of 2018. There were seven samples co-infected with H1N1 and Yamagata, whereas three samples were co-infected with both H1N1 and Victoria. No co-infections were detected at the same time within the past 2 years (2015/16 and 2016/17). H3N2 was the predominant influenza type before week 44 of 2017 (Fig. 1b). Seasonal activity continued with the co-circulation of H3N2 and B (Yamagata) virus between week 45 and week 47. The positive rate significantly increased until week 1 of 2018 with B (Yamagata) virus being the predominant subtype. The ratio of H1N1 increased from week 2 of 2018, after which H1N1 became predominant with B (Yamagata) co-circulation. H1N1 was predominant after week 5 of 2018 (Fig. 1b).

Positive sample rates for influenza (B virus and H1N1) peaked from week 46 in 2017 to week 12 in 2018 (Fig. 1b). There was also a sub-peak (mainly for H3N2) from week 38 to week 41 of 2017 (Fig. 1b). No obvious difference existed among the 11 prefecture-level cities regarding the positive sample rate (Supplementary Fig. S2b). The predominant strain was influenza A from week 36 to week 46 in 2017 (Fig. 1b). From about week 47 in 2017, the predominant strain changed to influenza B in most cities (Zhoushan and Taizhou being 4 weeks ahead, and Lishui having a 4-week lag) (Supplementary Fig. S2c). From approximately week 3 of 2018, influenza A and B both prevailed in Zhejiang Province. Most (82.97%) participants were children aged younger than 14 years (Supplementary Table S1).

A total of 57 influenza genomes were obtained during the 2017/18 period, including 13 samples of H1N1, eight samples of H3N2 and 36 samples of B virus (19 samples of Yamagata and 17 samples of Victoria). HA and NA sequences were found to share 96.2–99.0% identity at the nucleotide level with the vaccine strains and 90.8–98.5% at the amino acid level (Supplementary Table S3). We used strains composing the quadrivalent vaccines (http://www.who.int/influenza/vaccines/virus/recommendations/2017_18_north/en/) that were recommended by the WHO as reference sequences to infer substitutions in the obtained sequences. Several mutations were found in segments as follows (Supplementary Table S4). For the H1N1 isolates, there were four strains (A/Zhejiang-Nanxun/SWL115/2018, A/Zhejiang-Wuxing/SWL122/2018, A/Zhejiang-Yuecheng/SWL143/2018 and A/Zhejiang-Kecheng/SWL113/2018) with an HA 190 loop region (ILVLWGIHH) substitution, which was related to the human receptor binding site. I354L and V344M could modulate PB2 activity in snatching caps from host RNAs, whereas N321K of PA has been reported to enhance polymerase complex activity *in vitro* and virus replication in cell culture [3]. N121K substitution was found in two HA segments in H3N2 (A/Zhejiang-Xihu/35/2017 and A/Zhejiang-Jiaojiang/113/2018). This was one of the important amino acid substitutions in the epidemic strains isolated during the 2016/17 influenza season as an antigenic variant in H3N2. Both R158K in the antigenic epitope B and R277Q in the antigenic epitope C were also found in H3N2 HA. Two mutations in HA for B virus (Victoria lineage), V132I and D144N, were found in the 120 loop and 150 loop, respectively; these are major antigenic epitopes reported in previous studies [4 5].

The phylogenetic tree based on H1N1 HA demonstrated that the strains clustered into a large monophyletic clade with the vaccine strain, A/Michigan/45/2015 (Supplementary Fig. S3). Isolates obtained from severe patient cases were also grouped together, with a bootstrap value of 90. H1N1 isolates from the Zhejiang Province belonged to the genetic clade 6B.1 and were represented by globally circulating H1N1 (Qatar, HongKong, Shanghai in Mainland China, Supplementary Fig. S3). The phylogenetic tree based on H3N2 HA demonstrated the strains to be in the 3C.2 group, which is similar to the Canadian surveillance observations (83%) and recent reports from Europe [6]. These were grouped into one monophyletic clade with the vaccine strain A/Hongkong/4801/2014. H3N2 strains obtained in this study were not isolated from other regions of China from 2014 to 2017 (Supplementary Fig. S4). Phylogenetic analysis of HA in the B virus showed that isolates from Zhejiang Province from 2017 to 2018 were of two main lineages, Yamagata and Victoria (Supplementary Fig. S5). For the Yamagata lineage, sequences in this study were clustered into one monophyletic clade with a bootstrap value of 85. All available HA gene sequences of the B/Yamagata/16/88 lineage viruses belonged to the genetic clade 3. Sequences of the Victoria lineage were also in a monophyletic clade with a bootstrap value of 99. The HA gene sequences of the B/Victoria/2/87 lineage viruses belonged to the genetic clade 1A.

This study reported the co-circulation of influenza A and B viruses in Zhejiang Province, China, during the 2017–2018 flu season, including H1N1, H3N2, B(Yamagata) and B(Victoria). Cases of co-infection were also detected, which might frequently occur during the influenza season with a high occurrence of ILI. Most countries and regions also experienced simultaneous influenza A and B epidemics. B (Yamataga) was predominant in European countries, while H3N2 was predominant in the USA [7]. Different regions might have different predominant stains. Some studies reported that the influenza B virus comprised <10% of the detected influenza, whereas in 2017/18, the B virus was identified in equal proportion to influenza H3N2 [6]. A similar phenomenon was found in Zhejiang Province, China, where the influenza B virus epidemic was at a lower level (unpublished data) in both 2016/17 and 2015/16. Although the reasons for this influenza B (Yamagata) onset are unclear, we speculate the following reasons. On the one hand, with regards to vaccine mismatch and its effectiveness, the strains composing the trivalent vaccine administered in China during 2017/18 were those recommended by the WHO for influenza strains in the northern hemisphere (http://www.who.int/influenza/vaccines/virus/recommendations/2017_18_north/en/). Since the trivalent vaccine, rather than the quadrivalent vaccine (B Yamagata lineage was absent in the trivalent vaccine), was administered in Zhejiang Province, the Yamagata lineage of the B virus was likely responsible for the high level of epidemics in the region. Alternatively, the effectiveness of seasonal influenza vaccines can vary by season, based on the different circulating influenza viruses, including H1N1, H3N2 and B viruses (both the Yamagata and Victor lineages). It was reported that the overall estimated effectiveness of the 2017/18 seasonal influenza vaccine for the prevention of medically-attended, laboratory-confirmed influenza infection was 36% [8]. On the other hand, the influenza vaccination coverage in mainland China was low. The vaccination coverage rate was only 1.5–2% between 2004 and 2014 in a national survey [9]. The percentage in China continues to remain uncertain, since statistics regarding influenza vaccination has not been determined for 2017/18. The influencing factors of vaccination coverage included education level, knowledge, attitude towards the influenza vaccination policy, which were highly complicated [9]. However, vaccination continues to be recommended to reduce the risk of illness and serious complications. Furthermore, long-term surveillance remains essential as a means by which to provide an early warning to public health departments regarding the potential for viral circulation and transmission, and is a fundamental aspect of vaccine strain/composition recommendation.

These phylogenetic trees based on the HA segments from different influenza types and the topological structure of the monophyletic clade were in accordance with the conclusion that H1N1, H3N2 and B viruses were co-circulating in Zhejiang Province in 2017/18, which reflected the different type of flu virus evolution pattern in recent years. As an example, the H1N1 sequence from a district of Jiading in Shanghai(A/Shanghai-Jiading/1970/2015) showed high homology with both sequences from the city of Huzhou (A/Zhejiang-Nanxun/SWL115/2018 and A/Zhejiang-Wuxing/SWL122/2018) in Zhejiang (Supplementary Fig. S3). This may indicate an introduction event from Shanghai (outside of Zhejiang) to Huzhou (inside of Zhejiang) that played a role in influenza circulation and evolution dynamics, as has been reported previously [4 10]. Our previous study demonstrated that multiple viral introductions from both Chinese and international sources contributed to the B virus epidemic co-circulation in coastal southeastern China. Bahl *et al*. have stated that multiple geographic origins may seed influenza epidemics [10]. Zhejiang is a Province with a huge migratory population, which presents the opportunity for the introduction of influenza between regions. More samples and detailed epidemiology information are needed to comprehensively understand viral dynamics and the migration of influenza in this region.

In this study, we reported the co-circulation of influenza A and B viruses in Zhejiang Province, China, during the 2017/18 influenza season, including H1N1, H3N2, B (Yamagata) and B (Victoria), with a high level of ILI compared to the same period during the 2016/17 and 2015/16 seasons. To overcome the limitations of the present study and provide a more in-depth analysis in the future studies, the following should be considered: (1) information on how many individuals were vaccinated during this period and if there was any relationship with the number of influenza co-infections; and (2) more detailed information regarding the molecular epidemiology of influenza should be included in future studies.

## Supplementary material

For supplementary material accompanying this paper visit https://doi.org/10.1017/S0950268820000412. click here to view supplementary material
