# Supplementary figures and images for: Co-circulation of influenza A(H1N1), A(H3N2), B(Yamagata) and B(Victoria) during the 2017−2018 influenza season in Zhejiang Province, China
Source: Epidemiol Infect. 2020 Feb 14;148:e296. doi: 10.1017/S0950268820000412 (PMC7770466; doi:10.1017/S0950268820000412)

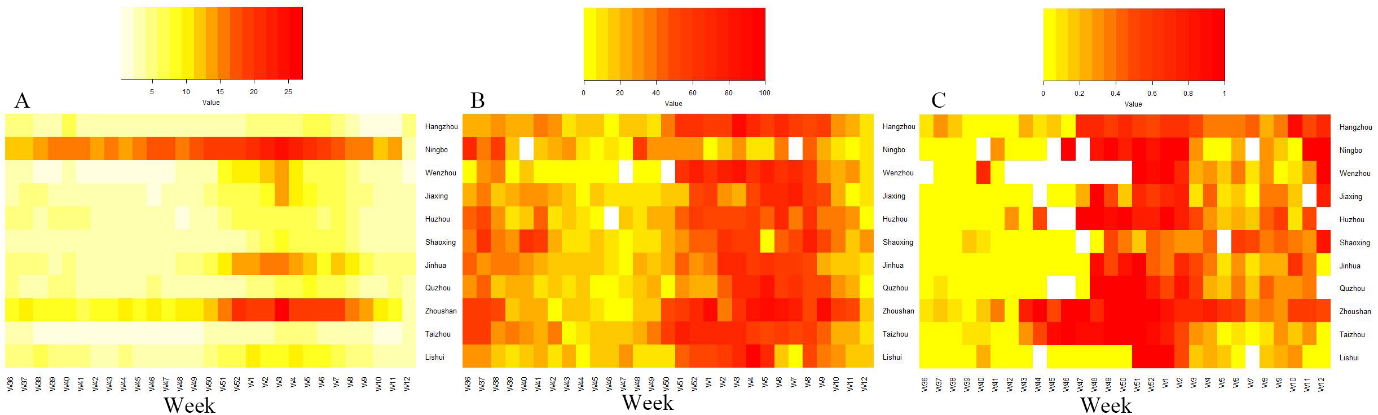

Supplement: Supplementary file 1 [file S0950268820000412sup001.zip › S0950268820000412sup001.docx]

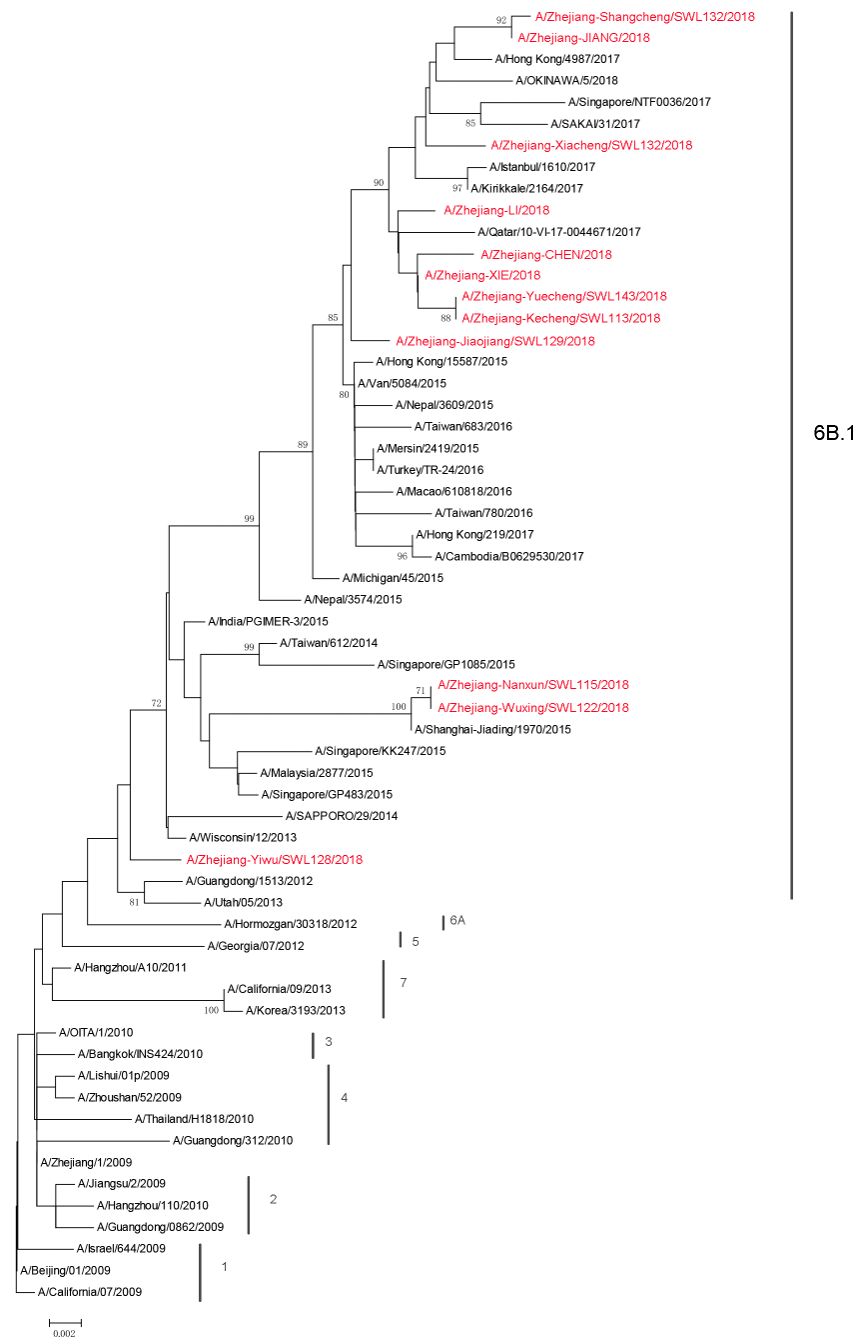

Supplement: Supplementary file 1 [file S0950268820000412sup001.zip › S0950268820000412sup002.png]

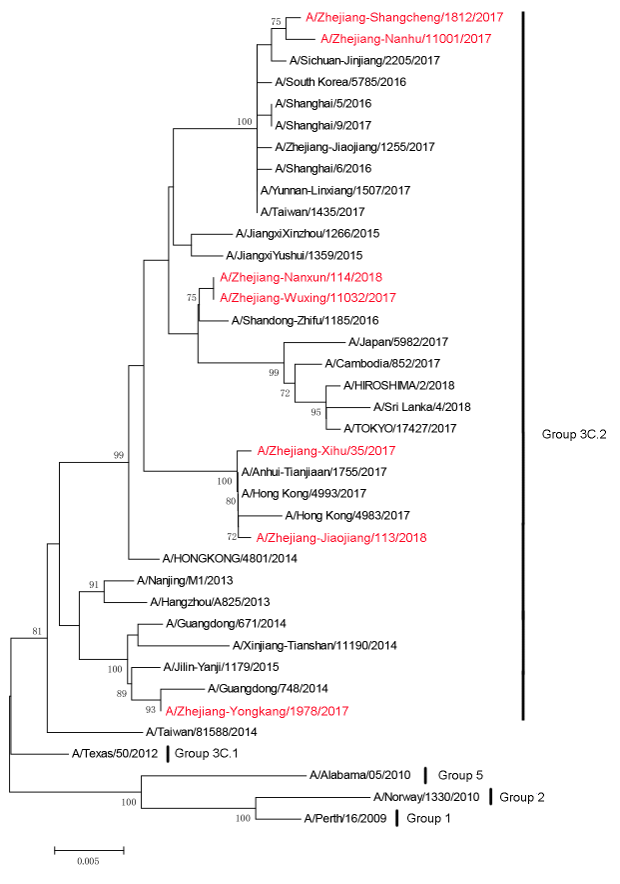

Supplement: Supplementary file 1 [file S0950268820000412sup001.zip › S0950268820000412sup003.png]

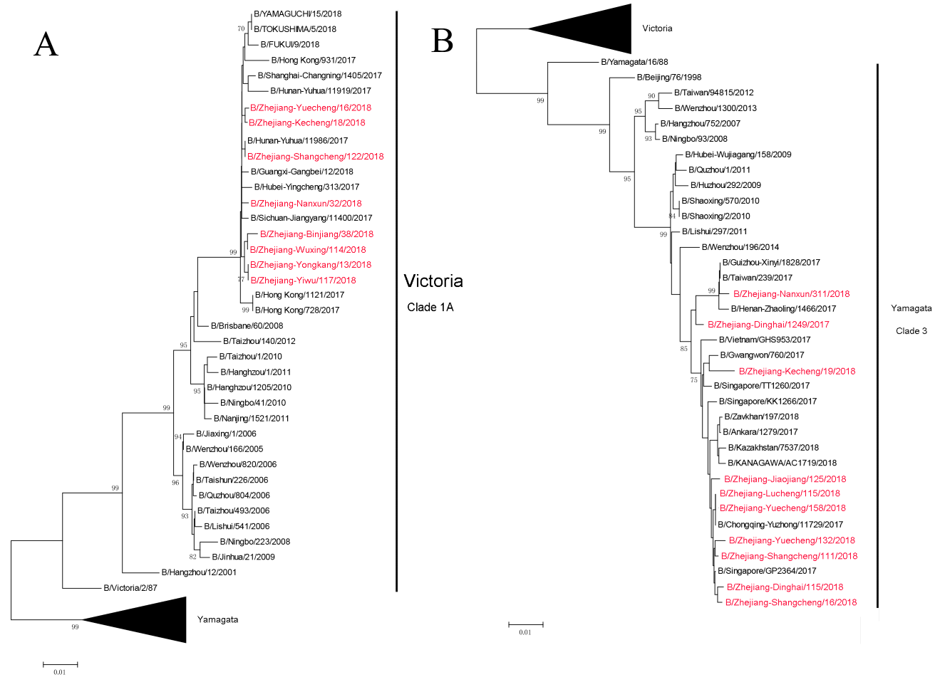

Supplement: Supplementary file 1 [file S0950268820000412sup001.zip › S0950268820000412sup004.png]
