# Supplementary material for: Co-circulation of influenza A(H1N1), A(H3N2), B(Yamagata) and B(Victoria) during the 2017−2018 influenza season in Zhejiang Province, China
Source: Epidemiol Infect. 2020 Feb 14;148:e296. doi: 10.1017/S0950268820000412 (PMC7770466; doi:10.1017/S0950268820000412)
Supplement: Supplementary file 1 [file S0950268820000412sup001.zip › S0950268820000412sup006.zip!/Supplementary material/Supplementary material.docx]

Supplementary material

Figure 1. Location of 16 surveillance sites in Zhejiang Province, China. Number listed is in accordance with the supplementary table 2 for the information on total outpatient visit number for each hospital in past 3 years.

Figure 2. Spatiotemporal series results represented by heat maps. A: Heat map of ILI% by prefecture-level cities from week 36, 2017 to week 12, 2018. B: Heat map of positive sample rates by prefecture-level cities from week 36, 2017 to week 12, 2018. The white block indicates that no sample was detected. C: Heat map of influenza B proportion in all positive samples by prefecture-level cities from week 36, 2017 to week 12, 2018. The white block indicates that no sample was detected.

Figure 3. Neighbor-joining tree of H1N1 based on HA segment. Red represents the isolates obtained in this study. Bootstrap values based on 1000 replicates are indicated above the branches.

Figure 4. Neighbor-joining tree of H3N2 based on HA segment. Red represents the isolates obtained in this study. Bootstrap values based on 1000 replicates are indicated above the branches.

Figure 5. Neighbor-joining tree of influenza B based on HA segment. Red represents the isolates obtained in this study. Bootstrap values based on 1000 replicates are indicated above the branches. A: Victoria lineage; B: Yamagata lineage.

Table 1. General information on ILI during the period from week 36 in 2015 to week 12 in 2016, week 36 in 2016 to week 12 in 2017, and week 36 in 2017 to week 12 in 2018.

| Period | Week | ILI number（age） | | | | | Total number of ILI | Total number of outpatient and emergency patient | ILI (%) |
| --- | --- | --- | --- | --- | --- | --- | --- | --- | --- |
|  |  | 0～ | 5～ | 15～ | 25～ | 60～ |  |  |  |
| 2015/16 | 201536 | 2140 | 599 | 92 | 338 | 109 | 3278 | 127042 | 2.58 |
|  | 201537 | 2137 | 624 | 93 | 265 | 84 | 3203 | 131488 | 2.44 |
|  | 201538 | 2134 | 542 | 71 | 230 | 79 | 3056 | 136491 | 2.24 |
|  | 201539 | 2536 | 701 | 112 | 241 | 79 | 3669 | 138371 | 2.65 |
|  | 201540 | 2319 | 563 | 66 | 233 | 66 | 3247 | 126470 | 2.57 |
|  | 201541 | 2196 | 561 | 64 | 218 | 72 | 3111 | 134722 | 2.31 |
|  | 201542 | 2549 | 762 | 78 | 218 | 72 | 3679 | 144585 | 2.54 |
|  | 201543 | 2521 | 773 | 82 | 202 | 70 | 3648 | 150794 | 2.42 |
|  | 201544 | 2426 | 683 | 78 | 253 | 76 | 3516 | 144033 | 2.44 |
|  | 201545 | 2340 | 735 | 76 | 172 | 67 | 3390 | 148423 | 2.28 |
|  | 201546 | 2254 | 804 | 68 | 237 | 80 | 3443 | 148262 | 2.32 |
|  | 201547 | 2710 | 727 | 95 | 258 | 84 | 3874 | 155198 | 2.50 |
|  | 201548 | 2446 | 699 | 49 | 252 | 84 | 3530 | 151693 | 2.33 |
|  | 201549 | 2645 | 781 | 85 | 248 | 57 | 3816 | 157398 | 2.42 |
|  | 201550 | 2834 | 907 | 92 | 281 | 81 | 4195 | 165739 | 2.53 |
|  | 201551 | 3062 | 939 | 102 | 296 | 81 | 4480 | 164666 | 2.72 |
|  | 201552 | 3013 | 1178 | 112 | 301 | 94 | 4698 | 167110 | 2.81 |
|  | 201553 | 3158 | 1417 | 106 | 408 | 105 | 5194 | 163973 | 3.17 |
|  | 201601 | 3444 | 1606 | 135 | 392 | 98 | 5675 | 170623 | 3.33 |
|  | 201602 | 3190 | 1989 | 129 | 433 | 109 | 5850 | 163455 | 3.58 |
|  | 201603 | 3037 | 1765 | 151 | 541 | 137 | 5631 | 142832 | 3.94 |
|  | 201604 | 2775 | 1202 | 173 | 602 | 153 | 4905 | 140261 | 3.50 |
|  | 201605 | 2313 | 891 | 124 | 549 | 141 | 4018 | 133936 | 3.00 |
|  | 201606 | 2433 | 890 | 147 | 745 | 203 | 4418 | 96218 | 4.59 |
|  | 201607 | 2800 | 790 | 132 | 546 | 164 | 4432 | 148222 | 2.99 |
|  | 201608 | 2581 | 918 | 184 | 653 | 155 | 4491 | 140782 | 3.19 |
|  | 201609 | 3401 | 1503 | 195 | 601 | 128 | 5828 | 151679 | 3.84 |
|  | 201610 | 3239 | 1601 | 175 | 478 | 108 | 5601 | 145299 | 3.85 |
|  | 201611 | 3603 | 2266 | 240 | 675 | 122 | 6906 | 155215 | 4.45 |
|  | 201612 | 3681 | 2261 | 151 | 534 | 124 | 6751 | 154243 | 4.38 |
| total |  | 81917 | 31677 | 3457 | 11400 | 3082 | 131533 | 4399223 | 2.99 |
| percentage (%) |  | 62.28 | 24.08 | 2.63 | 8.67 | 2.34 |  |  |  |
| 2016/17 | 201636 | 1724 | 496 | 87 | 341 | 107 | 2755 | 118970 | 2.32 |
|  | 201637 | 1812 | 534 | 88 | 298 | 92 | 2824 | 113214 | 2.49 |
|  | 201638 | 2364 | 690 | 112 | 309 | 77 | 3552 | 130628 | 2.72 |
|  | 201639 | 2159 | 617 | 99 | 372 | 88 | 3335 | 132633 | 2.51 |
|  | 201640 | 2206 | 640 | 94 | 287 | 65 | 3292 | 126259 | 2.61 |
|  | 201641 | 2319 | 724 | 123 | 362 | 88 | 3616 | 144139 | 2.51 |
|  | 201642 | 2634 | 906 | 148 | 366 | 84 | 4138 | 150412 | 2.75 |
|  | 201643 | 2989 | 1069 | 146 | 413 | 104 | 4721 | 156960 | 3.01 |
|  | 201644 | 3033 | 1288 | 180 | 485 | 109 | 5095 | 161108 | 3.16 |
|  | 201645 | 3200 | 1526 | 155 | 460 | 98 | 5439 | 161247 | 3.37 |
|  | 201646 | 3492 | 1840 | 203 | 590 | 141 | 6266 | 171538 | 3.65 |
|  | 201647 | 3215 | 1662 | 192 | 469 | 102 | 5640 | 161891 | 3.48 |
|  | 201648 | 3397 | 2245 | 203 | 558 | 111 | 6514 | 168170 | 3.87 |
|  | 201649 | 3559 | 2116 | 231 | 624 | 113 | 6643 | 177121 | 3.75 |
|  | 201650 | 3398 | 1854 | 162 | 481 | 90 | 5985 | 167709 | 3.57 |
|  | 201651 | 3423 | 1954 | 273 | 576 | 124 | 6350 | 168285 | 3.77 |
|  | 201652 | 3445 | 1861 | 260 | 560 | 143 | 6269 | 162857 | 3.85 |
|  | 201701 | 3229 | 1597 | 215 | 617 | 133 | 5791 | 160997 | 3.60 |
|  | 201702 | 2878 | 1302 | 185 | 540 | 152 | 5057 | 158829 | 3.18 |
|  | 201703 | 2686 | 1106 | 134 | 554 | 139 | 4619 | 158755 | 2.91 |
|  | 201704 | 2211 | 795 | 135 | 609 | 154 | 3904 | 119388 | 3.27 |
|  | 201705 | 2228 | 534 | 132 | 507 | 120 | 3521 | 118717 | 2.97 |
|  | 201706 | 2232 | 520 | 133 | 527 | 115 | 3527 | 146992 | 2.40 |
|  | 201707 | 2518 | 762 | 170 | 642 | 155 | 4247 | 148609 | 2.86 |
|  | 201708 | 2779 | 911 | 160 | 563 | 164 | 4577 | 150214 | 3.05 |
|  | 201709 | 3170 | 1418 | 203 | 720 | 139 | 5650 | 162640 | 3.47 |
|  | 201710 | 3072 | 1538 | 208 | 638 | 142 | 5598 | 165440 | 3.38 |
|  | 201711 | 3133 | 1726 | 179 | 596 | 139 | 5773 | 166131 | 3.47 |
|  | 201712 | 3058 | 1772 | 185 | 595 | 128 | 5738 | 165295 | 3.47 |
| total |  | 81563 | 36003 | 4795 | 14659 | 3416 | 140436 | 4395148 | 3.20 |
| percentage (%) | | 58.08 | 25.64 | 3.41 | 10.44 | 2.43 |  |  |  |
| 2017/18 | 201736 | 4031 | 1031 | 312 | 962 | 305 | 6641 | 141790 | 4.68 |
|  | 201737 | 4249 | 1256 | 253 | 801 | 233 | 6792 | 148731 | 4.57 |
|  | 201738 | 3907 | 1222 | 222 | 607 | 190 | 6148 | 162886 | 3.77 |
|  | 201739 | 4284 | 1187 | 260 | 627 | 214 | 6572 | 152777 | 4.30 |
|  | 201740 | 4603 | 1159 | 187 | 636 | 188 | 6773 | 126976 | 5.33 |
|  | 201741 | 3804 | 990 | 201 | 557 | 180 | 5732 | 149696 | 3.83 |
|  | 201742 | 3653 | 1010 | 158 | 438 | 149 | 5408 | 149239 | 3.62 |
|  | 201743 | 4124 | 1222 | 146 | 425 | 104 | 6021 | 162579 | 3.70 |
|  | 201744 | 4470 | 1190 | 148 | 452 | 103 | 6363 | 170020 | 3.74 |
|  | 201745 | 4370 | 1304 | 145 | 416 | 91 | 6326 | 173147 | 3.65 |
|  | 201746 | 4376 | 1237 | 115 | 393 | 102 | 6223 | 171450 | 3.63 |
|  | 201747 | 4324 | 1663 | 115 | 434 | 93 | 6629 | 170729 | 3.88 |
|  | 201748 | 4515 | 1753 | 176 | 474 | 121 | 7039 | 180217 | 3.91 |
|  | 201749 | 4669 | 2297 | 177 | 514 | 138 | 7795 | 185242 | 4.21 |
|  | 201750 | 4824 | 3349 | 280 | 644 | 169 | 9266 | 191693 | 4.83 |
|  | 201751 | 5541 | 5992 | 401 | 985 | 154 | 13073 | 200265 | 6.53 |
|  | 201752 | 7068 | 8052 | 634 | 1405 | 284 | 17443 | 219544 | 7.95 |
|  | 201801 | 7387 | 7094 | 664 | 1541 | 340 | 17026 | 200842 | 8.48 |
|  | 201802 | 8069 | 7238 | 757 | 1795 | 297 | 18156 | 200800 | 9.04 |
|  | 201803 | 10237 | 8310 | 1216 | 3056 | 509 | 23328 | 224895 | 10.37 |
|  | 201804 | 8015 | 5976 | 788 | 2420 | 442 | 17641 | 192132 | 9.18 |
|  | 201805 | 7520 | 4404 | 656 | 2328 | 434 | 15342 | 181209 | 8.47 |
|  | 201806 | 7388 | 3036 | 659 | 2323 | 419 | 13825 | 189145 | 7.31 |
|  | 201807 | 5850 | 1931 | 454 | 2104 | 480 | 10819 | 142035 | 7.62 |
|  | 201808 | 5143 | 1542 | 367 | 1967 | 467 | 9486 | 168498 | 5.63 |
|  | 201809 | 4523 | 1234 | 268 | 1394 | 374 | 7793 | 180915 | 4.31 |
|  | 201810 | 3401 | 1052 | 226 | 890 | 216 | 5785 | 163901 | 3.53 |
|  | 201811 | 3625 | 1133 | 211 | 753 | 137 | 5859 | 167559 | 3.50 |
|  | 201812 | 3380 | 1112 | 231 | 749 | 144 | 5616 | 165151 | 3.40 |
| total |  | 151350 | 79976 | 10427 | 32090 | 7077 | 280920 | 5034063 | 5.58 |
| percentage (%) | | 53.88 | 28.47 | 3.71 | 11.42 | 2.52 |  |  |  |

Table 2. Total outpatient visit number for each hospital in past 3 years

| Hospital | Period | | |
| --- | --- | --- | --- |
|  | 2015/16 | 2016/17 | 2017/18 |
| 1 | 367036 | 356552 | 393344 |
| 2 | 584098 | 624824 | 770325 |
| 3 | 140662 | 147465 | 127609 |
| 4 | 181774 | 179003 | 193002 |
| 5 | 744421 | 722226 | 825931 |
| 6 | 168122 | 189166 | 201720 |
| 7 | 315406 | 282972 | 328457 |
| 8 | 129983 | 137623 | 166347 |
| 9 | 382719 | 356710 | 386904 |
| 10 | 299452 | 291396 | 323094 |
| 11 | 177890 | 214900 | 251695 |
| 12 | 91493 | 100289 | 120321 |
| 13 | 137952 | 130200 | 146073 |
| 14 | 330542 | 332361 | 423186 |
| 15 | 278688 | 248943 | 294805 |
| 16 | 68985 | 73791 | 72950 |

Table 3 Information for each HA and NA segment for each type of influenza

| Influenza Type | Segment | NT (%) | AA (%) | Vaccine Strain Identity |
| --- | --- | --- | --- | --- |
| H1N1 | HA | 98.1 | 98.5 | A/Michigan/45/2015 |
|  | NA | 99.0 | 97.9 |  |
| H3N2 | HA | 98.6 | 98.0 | A/Hongkong/4801/2014 |
|  | NA | 98.6 | 97.4 |  |
| BV | HA | 98.8 | 97.1 | B/Brisbane/60/2008 |
|  | NA | 96.2 | 90.8 |  |
| BY | HA | 98.6 | 96.8 | B/Phuket/3073/2013 |
|  | NA | 97.9 | 95.1 |  |

NT: Nucleotide;

AA: Amino acid.

Table 4. Substitutions in segments in H1N1, H3N2 and B virus (Victoria). Substitutions that are consisted with each vaccine strain accordingly are not present in below.

| Influenza type | Segment | Substitution | Mutations responsible for phenotype change |
| --- | --- | --- | --- |
| H1N1 | HA | 190 loop (ILVLWGIHH) | Receptor Binding Site |
|  | PB2 | I135L & V344M | Modulate PB2 activity in snatching caps from host RNAs |
|  | PA | N321K | Enhance polymerase complex activity in vitro and virus replication in cell culture |
| H3N2 | HA | Cleavage site (PERQTRG) | High pathogen virus |
|  |  | N121K | Antigenic variation |
|  |  | R158K | Antigenic epitope B area |
|  |  | R277Q | Antigenic epitope C area |
| BV | HA | 120 loop (V132I) | Major antigenic epitope |
|  |  | 150 loop (D144S/N) | Major antigenic epitope |
